# Tobacco use and caries risk among adolescents – a longitudinal study in Sweden

**DOI:** 10.1186/1472-6831-13-31

**Published:** 2013-07-15

**Authors:** Anders Holmén, Ulf Strömberg, Kerstin Magnusson, Svante Twetman

**Affiliations:** 1 Department of Research and Development, Halland Hospital, SE-301 85 Halmstad, Sweden; 2 Department of Occupational and Environmental Medicine, Lund University, SE-221 85 Lund, Sweden; 3 Section of Community and Preventive Dentistry, Maxillofacial Unit,Halland Hospital, SE-301 85 Halmstad, Sweden; 4 Department of Cariology, Endodontics, Pediatric Dentistry and Clinical Genetics, Institute of Dentistry, Faculty of Health and Medical Sciences, University of Copenhagen, Nørre Allé 20, 2200 Copenhagen N, Denmark

**Keywords:** Adolescents, Caries, Tobacco use, Socio-economy, Prevention

## Abstract

**Background:**

Smoking and the use of smokeless tobacco have a detrimental impact on general and oral health. The relationship to dental caries is however still unclear. As caries is a multi-factorial disease with clear life-style, socio-economic and socio-demographic gradients, the tobacco use may be a co-variable in this complex rather than a direct etiological factor. Our aim was to analyze the impact of tobacco use on caries incidence among adolescents, with consideration to socio-economic variables by residency, using epidemiological data from a longitudinal study in the region of Halland, Sweden.

**Methods:**

The study population consisted of 10,068 adolescents between 16–19 years of age from whom yearly data on caries and tobacco use (cigarette smoking and use of smokeless tobacco) were obtained during the period 2006–2012. Reported DMFS increment between 16 and 19 years of age (∆DMFS) for an individual was considered as the primary caries outcome. The outcome data were compared for self-reported never vs. ever users of tobacco, with consideration to neighborhood-level socio-economy (4 strata), baseline (i.e., 16 years of age) DMFS and sex. The region consists of 65 parishes with various socio-economic conditions and each study individual was geo-coded with respect to his/her residence parish. Neighborhood (parish-level) socio-economy was assessed by proportion of residing families with low household purchasing power.

**Results:**

∆DMFS differed evidently between ever and never users of tobacco (mean values: 1.8 vs. 1.2; proportion with ∆DMFS > 0: 54.2% vs. 40.5%; p < 0.0001). Significant differences were observed in each neighborhood-level socio-economic stratum. Even after controlling for baseline DMFS and sex, ∆DMFS differed highly significantly between the ever and never users of tobacco (overall p < 0.0001).

**Conclusion:**

Tobacco use was clearly associated with increased caries increment during adolescence. Hence, this factor is relevant to consider in the clinical caries risk assessment of the individual patient as well as for community health plans dealing with oral health.

## Background

It is generally thought that smoking and the use of smokeless tobacco may have a detrimental impact on general and oral health [1-3]. The relationship to dental caries is however still unclear. Several studies world-wide have denominated tobacco use as a risk factor for coronal and root caries and disclosed increased caries rates in tobacco smoking young adults, adults, and elderly [4-9]. However, a recent epidemiologic survey conducted in Sweden failed to demonstrate a relationship between tobacco use and caries in adults and elderly [10]. A recent systematic review on tobacco use and dental caries was based solely on cross-sectional studies and, as a result, the overall quality of evidence was concluded to be poor [11].

As caries is a multi-factorial disease with clear life-style, socio-economic and socio-demographic gradients, the tobacco use may be a co-variable in this complex rather than a direct etiological factor [12,13]. We have previously presented caries risk in children and adolescents in relation to neighborhood socio-economic factors with aid of a geo-mapping tool in the Region of Halland, Sweden [14,15]. The epidemiological data collected in this region provides an opportunity to examining the impact of tobacco use on caries risk among adolescents, with consideration to socio-economic variables by residency. Importantly, as the epidemiological data have been collected annually, we can apply a longitudinal approach.

## Methods

### Study population

The vast majority of all children and adolescents (93%) in the region are listed as regular patients at the Public Dental Service that provides free dental care between 1 and 19 years with recall intervals varying from 3 to 24 months depending on the individual need. Data on the experience of manifest (dentin) caries is registered according to the WHO-criteria [16] and annually reported to the community dentistry unit. Caries data were primarily based on clinical examinations and bitewing radiographs were only taken on individual indications.

Annual data on caries and tobacco use were collected during the period 2006–2012. We considered four birth-year-cohorts, 1990, 1991, 1992 and 1993, with observation periods 2006–2009, 2007–2010, 2008–2011 and 2009–2012, respectively. Reported DMFS increment (∆DMFS) between 16 and 19 years of age, i.e. between the first and last year of the relevant observation period for an individual, was considered as the primary caries outcome. In total, the present study included 10,068 individuals with outcome data. The coverage of the total 16-19-year population of the Halland region was around 70%. The remaining adolescents were not recalled for a regular check-up at a relevant year or visited a private dentist at a clinic not among the in-reporting ones or located outside the region.

The study was approved by the Halland Hospital Ethical committee as well as The Swedish Data Inspection Board.

### Data on tobacco use

Each study individual was asked about his/her tobacco use at the annual clinical examination by the examining dentist or dental hygienist. Individuals who reported use of tobacco in at least at one of the four examination occasions were defined as ever users (n=1,459). Tobacco use included both cigarette smoking and the use of smokeless tobacco (snuff). Among those who reported use of tobacco, approximately 35% reported only use of snuff. No information on the amount or frequency of the tobacco use was available. All other individuals were classified as never users (n=8,609).

### Neighborhood socio-economy

**Figure 1:**
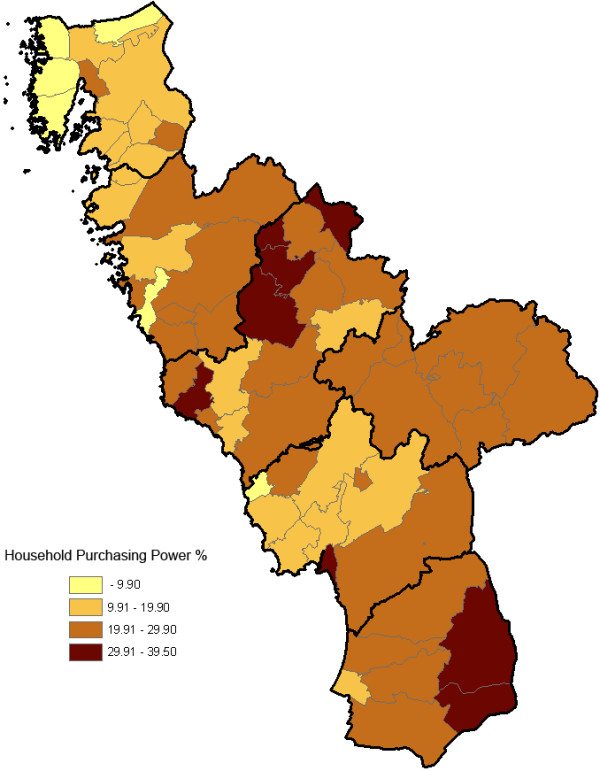
**Geo-map on household purchasing power for the 61 parishes in the county of Halland.** The residential areas (parishes) were classified into <10%, 10–19.9%, 20–20.9% and ≥30% of the residing families with low household purchasing power according to Swedish standards (see definition in the methods section). The thicker borderlines delimit the six municipalities of Halland.

Each study individual was geo-coded with respect to his/her residence parish. Statistics Sweden provided parish-level data from year 2010 on the socio-economic indicator we considered, viz. the proportion of families with low household purchasing power (according to Swedish standard; ≤19,500 USD household purchasing power) among all residing families with at least one child (≤19 years old; family with the same residence address). Household purchasing power was defined as total family disposable income adjusted for the composition of the family (number of adults and children). The parishes were classified into <10%, 10–19.9%, 20–20.9% and ≥30% based on this indicator (Figure 1).

### Statistical methods

The DMFS variable at a specific observation year (i.e., year of age for a given birth-year-cohort) was summarized by mean, significant caries index (SiC) based on DMFS [17] and proportion of caries-free (CF). The outcome variable ∆DMFS was summarized by mean, ∆SiC and proportion with ∆DMFS > 0. Outcome data for never vs. ever users of tobacco were basically compared by the Wilcoxon-Mann–Whitney test, as data were highly skewed, with a high proportion of “zero”. The complete data were generally stratified by birth-year and an overall *p*-value was obtained by the stratified Wilcoxon-Mann–Whitney test [18]. Moreover, further controlled overall p-values were computed by the stratified Wilcoxon-Mann–Whitney test (never vs. ever users) with additional stratification on neighborhood-level socio-economy, baseline DMFS (0, 1–3, >3) and sex. The cut-off values for baseline DMFS were based on the epidemiological caries data in the Halland region, reflecting no, low and high caries experience. IBM SPSS 20.0.2 and StatXact 6.2.0 (Cytel Inc., Cambridge, MA, USA) were used for the statistical analyses.

## Results

∆DMFS differed evidently between ever and never users of tobacco (Table 1; overall p < 0.0001). Significant differences were observed in each neighborhood-level socio-economic stratum (Table 2; overall p < 0.0001). As expected, a socio-economic gradient in caries burden was evident in the study population (Table 2). Nevertheless, baseline DMFS was consistently higher among ever users, as compared with never users in neighborhoods with a similar socio-economic characteristic (Table 2). Even after controlling for baseline DMFS and sex, ∆DMFS differed highly significantly between ever and never users (Table 3; overall p < 0.0001). The pattern of more pronounced DMFS increment during adolescence among the ever users of tobacco was consistent across the strata of neighborhood-level socio-economy and baseline DMFS, although the degree of statistical evidence varied (Table 3). The change in DMFS displayed a different pattern in the most vulnerable groups (SE groups 20–29.9 and 30+, baseline DMFS > 3), indicating a weaker influence of tobacco use. On the other hand, the statistical evidence of a tobacco effect was also weaker for the individuals in the residential areas with < 10% of the families with low purchasing power, within each stratum of baseline DMFS.

**Table 1:** DMFS outcome data at baseline and change in DMFS during the follow-up period for each tobacco group and birth cohort, with p-values for the tobacco group comparisons of change in DMFS

**Birth cohort**	**Tobacco group**	**DMFS baseline 16 years**				**Change in DMFS, 16–19 years**				
		**N**	**Mean**	SiC*	**CF†**	**N**	**ΔMean**	**ΔSiC**	**ΔDMFS>0%‡**	**p-value§**
1990	Ever users	390	3.7	7.7	27.7	390	1.8	4.2	57.2	< 0.001
	Never users	2233	2.9	7.6	35.9	2233	1.3	4.1	43.9	
1991	Ever users	342	3.9	8.3	25.4	342	2.0	4.5	57.3	< 0.001
	Never users	2191	2.6	7.5	39.4	2191	1.3	4.5	40.9	
1992	Ever users	357	3.5	8.0	31.1	357	1.8	4.6	56.0	< 0.001
	Never users	2070	2.7	7.6	38.4	2070	1.2	4.1	41.6	
1993	Ever users	370	3.5	7.8	33.5	370	1.6	4.6	46.5	< 0.001
	Never users	2116	2.5	7.3	41.0	2116	0.9	3.9	35.3	
Total	Ever users	1459	3.6	7.9	29.5	1459	1.8	4.5	54.2	< 0.001
	Never users	8609	2.7	7.5	38.7	8610	1.2	4.2	40.5	

* Significant caries index based on DMFS.

† Proportion caries-free.

‡ Proportion with new caries during the observation period (16–19 years).

§ P-value from the Wilcoxon-Mann–Whitney test comparing the distributions of DMFS between ever vs. never users.

The overall p-value < 0.0001 (stratified Wilcoxon-Mann–Whitney test).

**Table 2:** DMFS outcome data at baseline and change in DMFS during the follow-up period for each tobacco group and residential-area household purchasing power group, with p-values for the tobacco group comparisons of change in DMFS

**% low household purchasing power**	**Tobacco group**	**DMFS baseline 16 years**				**Change in DMFS, 16–19 years**				
		**N**	**Mean**	SiC*	**CF†**	**N**	**ΔMean**	**ΔSiC**	**ΔDMFS>0%‡**	**p-value§**
< 10	Ever users	152	2.5	7.6	41.4	152	1.3	4.2	43.4	0.02
	Never users	1553	1.7	6.5	49.8	1553	0.9	3.7	34.8	
10-19.9	Ever users	382	3.3	7.3	30.1	382	1.6	4.2	51.8	< 0.001
	Never users	2295	2.4	6.8	38.1	2295	1.0	3.8	38.2	
20-29.9	Ever users	755	3.9	8.0	27.3	755	1.8	4.6	56.7	< 0.001
	Never users	3826	3.0	7.8	35.8	3826	1.5	4.4	43.0	
30+	Ever users	162	4.4	8.9	27.8	162	2.3	5.0	59.3	< 0.001
	Never users	892	3.7	8.8	32.2	892	1.5	4.6	46.0	

* Significant caries index based on DMFS.

† Proportion caries-free.

‡ Proportion with new caries during the observation period (16–19 years).

§ P-value from the Wilcoxon-Mann–Whitney test comparing the distributions of DMFS between ever vs. never users.

The overall p-value < 0.0001 (stratified Wilcoxon-Mann–Whitney test).

**Table 3:** DMFS outcome data at baseline and change in DMFS during the follow up period for each tobacco group, residential-area household purchasing power group and baseline DMFS group, with p-values for the tobacco group comparisons of change in DMFS

			**DMFS baseline**		**ΔDMFS**		
**SE group**	**Baseline DMFS group**	**Tobacco group**	**N**	**Mean**	**N**	**Mean**	p-value*
< 10	0	Ever users	63	0.0	63	0.4	0.33
		Never users	774	0.0	774	0.4	
	1-3	Ever users	49	1.6	49	1.2	0.44
		Never users	492	1.7	492	1.0	
	> 3	Ever users	40	7.6	40	2.8	0.20
		Never users	287	6.5	287	1.8	
10 – 19.9	0	Ever users	115	0.0	115	0.7	0.05
		Never users	875	0.0	875	0.6	
	1-3	Ever users	129	1.9	129	1.6	0.001
		Never users	810	1.8	810	0.9	
	> 3	Ever users	138	7.3	138	2.5	0.002
		Never users	610	6.8	610	1.7	
20 – 29.9	0	Ever users	206	0.0	206	0.7	0.001
		Never users	1370	0.0	1370	0.5	
	1-3	Ever users	241	1.9	241	1.8	0.001
		Never users	1285	1.8	1285	1.1	
	> 3	Ever users	308	8.0	308	2.7	0.45
		Never users	1171	7.8	1171	2.5	
30+	0	Ever users	45	0.0	45	0.8	0.10
		Never users	287	0.0	287	0.6	
	1-3	Ever users	47	2.0	47	2.3	< 0.001
		Never users	293	1.9	293	1.2	
	> 3	Ever users	70	8.9	70	3.3	0.57
		Never users	312	8.8	312	2.6	

* P-value from the Wilcoxon-Mann–Whitney test comparing the distributions of DMFS between ever vs. never users.

The overall p-value < 0.0001 (stratified Wilcoxon-Mann–Whitney test; with additional stratification by sex).

## Discussion

To our knowledge, this report is based on the first longitudinal study addressing the impact of tobacco use on caries development among adolescents. The main finding was clear-cut; self-reported use of tobacco was significantly associated with increased incidence of dental caries over a 3 year-period, irrespective of socio-economic and geographic characteristics. Therefore, the immediate clinical implication is that the question “do you use tobacco?” is important and should be relevant to include as a component of the individual caries risk assessment. Such a comprehensive risk assessment is crucial for appropriate treatment decisions concerning prevention, non-operative and operative caries therapy [19-21] and for determination of individual recall intervals [22]. On population level, information on the use of tobacco should be incorporated in oral health community plans.

**Figure 2:**
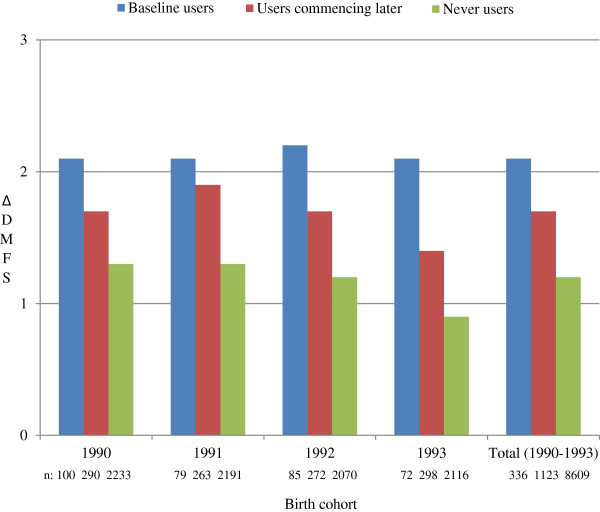
Caries development, depicted by mean values of ∆DMFS for each birth cohort as well as the total study group, for three exposure groups: reported tobacco users at baseline, i.e., 16 years of age (“baseline users”), the remaining users reported tobacco use at any subsequent occasion during the follow-up, i.e., 17–19 years of age (“users commencing later”) and “never users”, respectively.

The large size of the material, the high population coverage and the robust clinical scoring of caries were strongly enhancing the external validity of this project. The study design had still a number of limitations. The findings were based on a very crude classification of self-reported data on tobacco use, dichotomized as “ever“or “never” and the actual frequency and duration of the tobacco use was not reported. It is also likely that the true frequency of tobacco-use was somewhat under-reported, especially in the younger age-groups, due to reluctance to admit “a bad habit” in front of health professionals and sometimes parents. Furthermore, tobacco-use included both cigarette smoking and the use of smokeless tobacco (snuff) with differences in gender- and geographical pattern. Girls in urban parishes were more likely to smoke while the use of smokeless tobacco was more prevalent among rural boys (data not reported). In spite of exposure misclassification, likely to have induced bias towards the null, we were still able to demonstrate clear impact of reported tobacco use. Nevertheless, timing of exposure is an interesting aspect to investigate further. By dividing the “ever users” group into reported tobacco users at baseline (i.e., 16 years of age) and the remaining users reported tobacco use at any subsequent occasion during the follow-up (i.e., 17–19 years of age), our crude data indicate a higher mean ∆DMFS among the “baseline users” as compared with the “users commencing later” (Figure 2). Hence, the adverse effect on caries development could have been influenced by exposure time.

Another study limitation was that we were unable to control for other life-style and behavior-related factors although the adjustment for baseline DMFS and sex to some extent may have captured such obvious confounders.

The socio-economic statistics applied here were from 2010, but this was only a minor concern as the neighborhood characteristics proved to be stable over the total observation period [14]. Moreover, we considered family purchasing power as the primary indicator of neighborhood socio-economy. This indicator takes solely residing families with at least one child (≤19 years of age) into account; the elderly population was disregarded, which can be advocated. Nevertheless, family purchasing power corresponded fairly well with other alternative socio-economic indicators on the parish level, such as educational level and the proportion of immigrants [14].

Interestingly, the caries burden remained relatively unchanged over the four cohorts. On the other hand, the change in DMFS clearly depended on vulnerability (socioeconomic status), as seen in Table 3. A notable observation was that the SiC-index displayed less pronounced differences in caries incidence between tobacco-users and non-users compared with the crude mean values, considering the individuals in the neighborhoods with less favorable socio-economy (Table 2). As the distribution of caries is skewed, the SiC-index was constructed to more accurately mirror the dental health in the proportion of the population with the highest caries burden [17]. Consequently, those individuals were the adolescents with highest caries-activity and most filled surfaces at baseline, which may have limited the number of actual surfaces available for new obvious caries lesions over the study period. Bitewing radiographs were only taken on individual indications and were likely more often exposed in the high caries-group; this procedure may have enhanced the difference between the exposure groups. We also noticed that the statistical evidence of a tobacco effect was weaker for the individuals in the neighborhoods with most favorable socio-economy, within each stratum of baseline DMFS. Possible explanations could be a higher general awareness of factors associated with a healthy lifestyle or a less heavy use of tobacco products.

## Conclusion

In conclusion, tobacco use was clearly associated with increased caries development during adolescence. Thus, this factor should be relevant to include in the clinical caries risk assessment of the individual patient as well as for community health plans dealing with oral health.

## Competing interests

The authors have no conflicts of interests to declare.

## Authors’ contributions

All of the listed authors contributed to the conduct of the study. AH, US and ST analyzed/interpreted the data and drafted the manuscript. KM provided technical and administrative support. All authors approved the final version of this manuscript.

## Pre-publication history

The pre-publication history for this paper can be accessed here:

http://www.biomedcentral.com/1472-6831/13/31/prepub

## References

[B1] Critchley JA Unal B Health effects associated with smokeless tobacco: a systematic review Thorax 2003 58 435 443 10.1136/thorax.58.5.435 12728167 PMC1746661

[B2] Hanioka T Ojima M Tanaka K Matsuo K Sato F Tanaka H Causal assessment of smoking and tooth loss: a systematic review of observational studies BMC Public Health 2011 11 221 10.1186/1471-2458-11-221 21477320 PMC3087682

[B3] Nakamura K Huxley R Ansary-Moghaddam A Woodward M The hazards and benefits associated with smoking and smoking cessation in Asia: a meta-analysis of prospective studies Tob Control 2009 18 345 353 10.1136/tc.2008.028795 19617218

[B4] Matthews DC Clovis JB Brillant MG Filiaggi MJ McNally ME Kotzer RD Lawrence HP Oral health status of long-term care residents-a vulnerable population J Can Dent Assoc 2012 78 c3 22364866

[B5] Al-Habashneh R Al-Omari MA Taani DQ Smoking and caries experience in subjects with various form of periodontal diseases from a teaching hospital clinic Int J Dent Hyg 2009 7 55 61 10.1111/j.1601-5037.2008.00349.x 19215312

[B6] Campus G Cagetti MG Senna A Blasi G Mascolo A Demarchi P Strohmenger L Does smoking increase risk for caries? a cross-sectional study in an Italian military academy Caries Res 2011 45 40 46 10.1159/000322852 21228593

[B7] Rooban T Vidya K Joshua E Rao A Ranganathan S Rao UK Ranganathan K Tooth decay in alcohol and tobacco abusers J Oral Maxillofac Pathol 2011 15 14 21 10.4103/0973-029X.80032 21731272 PMC3125650

[B8] Tanaka K Miyake Y Arakawa M Sasaki S Ohya Y Household smoking and dental caries in schoolchildren: the Ryukyus Child Health Study BMC Public Health 2010 10 335 10.1186/1471-2458-10-335 20540808 PMC2893097

[B9] Bloom B Adams PF Cohen RA Simile C Smoking and oral health in dentate adults aged 18–64 NCHS Data Brief 2012 1 8 22617703

[B10] Hugoson A Hellqvist L Rolandsson M Birkhed D Dental caries in relation to smoking and the use of Swedish snus: epidemiological studies covering 20 years (1983–2003) Acta Odontol Scand 2012 70 289 296 10.3109/00016357.2011.654247 22339319

[B11] Benedetti G Campus G Strohmenger L Lingstrom P Tobacco and dental caries: a systematic review Acta Odontol Scand 2013 71 363 371 10.3109/00016357.2012.734409 23088732

[B12] Hanioka T Ojima M Tanaka K Yamamoto M Does secondhand smoke affect the development of dental caries in children? A systematic review Int J Environ Res Public Health 2011 8 1503 1519 10.3390/ijerph8051503 21655133 PMC3108123

[B13] Cinar AB Christensen LB Hede B Clustering of obesity and dental caries with lifestyle factors among Danish adolescents Oral health & preventive dentistry 2011 9 123 130 21842014

[B14] Stromberg U Holmén A Magnusson K Twetman S Geo-mapping of time trends in childhood caries risk–a method for assessment of preventive care BMC Oral Health 2012 12 9 10.1186/1472-6831-12-9 22510486 PMC3474168

[B15] Stromberg U Magnusson K Holmén A Twetman S Geo-mapping of caries risk in children and adolescents - a novel approach for allocation of preventive care BMC Oral Health 2011 11 26 10.1186/1472-6831-11-26 21943023 PMC3198761

[B16] Oral health surveys - basic methods World Health Organization 1997 Geneva: 4th Edition

[B17] Campus G Solinas G Maida C Castiglia P The 'Significant Caries Index' (SiC): a critical approach Oral health & preventive dentistry 2003 1 171 178 15641494

[B18] van Elteren PH On the combination of independent two sample tests of Wilcoxon B Int Statist Inst 1960 37 351 361

[B19] Young DA Featherstone JD Roth JR Anderson M Autio-Gold J Christensen GJ Fontana M Kutsch VK Peters MC Simonsen RJ Wolff MS Caries management by risk assessment: implementation guidelines Journal of the California Dental Association 2007 35 799 805 18080486

[B20] Pitts N Melo P Martignon S Ekstrand K Ismail A Caries risk assessment, diagnosis and synthesis in the context of a European Core Curriculum in Cariology European journal of dental education : official journal of the Association for Dental Education in Europe 2011 15 Suppl 1 23 31 22023543 10.1111/j.1600-0579.2011.00711.x

[B21] Twetman S Fontana M Featherstone J Caries risk assessment – can we achieve consensus? Community Dent Oral Epidemiol 2013 41 e64 e70 10.1111/cdoe.12026 24916679

[B22] Patel S Bay RC Glick M A systematic review of dental recall intervals and incidence of dental caries Journal of the American Dental Association 2010 141 527 539 20436100 10.14219/jada.archive.2010.0225

